# *ApoE* e4e4 Genotype and Mortality With COVID-19 in UK Biobank

**DOI:** 10.1093/gerona/glaa169

**Published:** 2020-07-04

**Authors:** Chia-Ling Kuo, Luke C Pilling, Janice L Atkins, Jane A H Masoli, João Delgado, George A Kuchel, David Melzer

**Affiliations:** 1 Connecticut Convergence Institute for Translation in Regenerative Engineering, University of Connecticut Health, Farmington; 2 Center on Aging, University of Connecticut Health, Farmington; 3 Epidemiology and Public Health Group, University of Exeter Medical School, UK; 4 Department of Healthcare for Older People, Royal Devon and Exeter Hospital, UK

We previously reported that the *ApoE* e4e4 genotype was associated with COVID-19 test positivity (odds ratio [OR] = 2.31, 95% CI: 1.65–3.24, *p* = 1.19 × 10^−6^) (1) in the UK Biobank (UKB) cohort, during the epidemic peak in England, from March 16 to April 26, 2020 (2). With more COVID-19 test results (March 16 to May 31, 2020) and mortality data (to March 31, 2020, with incomplete data for April 2020) linked to UKB, we reevaluated the *ApoE* e4 allele association with COVID-19 test positivity, and with all-cause mortality following test-confirmed COVID-19.

We restricted analyses to European-ancestry participants (3) (*n* = 451 367, 90% of sample) attending baseline assessment centers in England (*n* = 398 073) and excluded participants who died before the pandemic (set at February 1, 2020, *n* = 22 384). Single-nucleotide polymorphism data for rs429358 and rs7412 were used to determine *ApoE* genotypes. Our outcomes of interest were: (a) COVID-19 test positive versus the rest of the sample meeting inclusion criteria (ie, including untested samples and tested negative), and (b) tested positive and died versus the rest of the sample as above, but with additional exclusion of test positive participants who survived. Some of the excluded samples could have died but cannot be identified due to incomplete mortality data for April 2020. Logistic regression models compared *ApoE* e4e4 participants (or e3e4s) to e3e3s with adjustment for sex; age on April 26 or age at death; baseline UKB assessment center in England (accounting for geographical differences in viral exposures); genotyping array type; and the top five genetic principal components (accounting for possible population admixture).

The mean attained age was 68.2 years (*SD* = 8.0) with 174 667 females (55%). Of 219 747 e3e3 participants, 663 participants tested positive (302 per 100 000), of whom, 79 later died. Similarly, of 8767 e4e4 participants, 59 tested positive (673 per 100 000), of whom 13 later died (Table 1). In logistic models, *ApoE* e4e4 genotype was associated with increased risks of test positivity (OR = 2.24, 95% CI: 1.72–2.93, *p* = 3.24 × 10^−9^) and of mortality with test-confirmed COVID-19 (OR = 4.29, 95% CI: 2.38–7.72, *p* = 1.22 × 10^−6^), compared to e3e3s. For e3e4s versus e3e3s, these two associations were nominally statistically significant (at *p* < .05), but with much smaller effect sizes. The e4e4 associations were similar after excluding 50 566 participants related to the third degree or closer for test positivity (e4e4 OR = 2.30, 95% CI: 1.73–3.07, *p* = 1.39 × 10^−8^) and for mortality with test-confirmed COVID-19 (e4e4 OR = 4.53, 95% CI: 2.39–8.61, *p* = 3.87 × 10^−6^). Additionally, the e4e4 association with either COVID-19 outcome was little changed after removing participants with diseases associated with *ApoE* e4 alleles (4) and COVID-19 severity (5), including dementia, hypertension, coronary artery disease (myocardial infarction or angina), or type 2 diabetes (Table 1), based on diagnoses recorded from baseline self-reports and hospital discharge records during follow-up to March 2017. *ApoE* e3e4s were modestly associated with test positivity overall, and the association tended to be less marked in disease-free samples (Table 1). In additional analyses, we tested associations with *ApoE* e2 alleles, which have been linked to beneficial health outcomes (4). No associations were found between e2e3 and either of our COVID-19 outcomes (*p* > .05, vs e3e3). Analyses for e2e2s associations were underpowered (*n* = 2427, 4 positives, and 1 positive death).

**Table 1.** Risk of COVID-19 Test Positivity and Mortality, Comparing Participants With *ApoE* e3e4 or e4e4 to e3e3 Genotypes, in UK Biobank

							COVID-19 Positive vs Rest of Study Sample		COVID-19 Positive and Died vs Rest^†^ of Study Sample	
	*n*	Negative or Untested	Positive	Positive and Dead	Positivity Rate per 10^5^	Positivity and Death Rate per 10^5^	OR (95% CI)*	*p* Value	OR (95% CI)*	*p* Value
All										
e3e3	219 747	219 084	663	79	302	36				
e3e4	88 882	88 561	321	42	361	47	1.20 (1.05, 1.37)	.009	1.35 (0.92, 1.96)	.121
e4e4	8767	8708	59	13	673	148	2.24 (1.72, 2.93)	3.24E-9	4.29 (2.38, 7.72)	1.22E-6
Excluding dementia										
e3e3	219 392	218 744	648	76	295	35				
e3e4	88 558	88 263	295	34	333	39	1.13 (0.98, 1.29)	.093	1.14 (0.76, 1.70)	.536
e4e4	8676	8618	58	13	669	151	2.27 (1.74, 2.98)	2.42E-9	4.53 (2.51, 8.16)	5.21E-7
Excluding hypertension										
e3e3	147 332	146 958	374	31	254	21				
e3e4	59 655	59 483	172	17	288	29	1.13 (0.94, 1.35)	.186	1.39 (0.77, 2.51)	.278
e4e4	5918	5881	37	5	625	85	2.45 (1.75, 3.44)	2.10E-7	4.25 (1.65, 10.95)	.003
Excluding coronary artery disease										
e3e3	201 003	200 435	568	62	283	31				
e3e4	80 850	80 590	260	33	322	41	1.14 (0.98, 1.32)	.090	1.36 (0.89, 2.08)	.153
e4e4	7973	7923	50	10	627	126	2.23 (1.67, 2.98)	6.21E-8	4.23 (2.16, 8.26)	2.43E-5
Excluding type 2 diabetes										
e3e3	208 374	207 795	579	61	278	29				
e3e4	84 620	84 342	278	30	329	36	1.18 (1.02, 1.36)	.024	1.24 (0.80, 1.92)	.338
e4e4	8 391	8336	55	12	655	144	2.36 (1.79, 3.12)	1.23E-9	5.05 (2.72, 9.39)	3.08E-7

*Notes*: CI = confidence interval; OR = odds ratio.

*Adjusted for sex, age at death or age on April 26, 2020 (the last date of death), assessment center in England, genotyping array type, and the top five genetic principal components.

^†^Comparison group excluded participants testing positive and surviving.

The results presented imply a recessive effect of the *ApoE* e4 allele. Only modest associations were present between the much more common e3e4 genotype and COVID-19 outcomes, similar to results for rs429358 (which separates 0, 1, and 2 copies of e4 alleles, OR = 1.3, *p* = .0026) reported for severe COVID-19 with respiratory failure in a recent additive effect genome-wide analysis (6). *ApoE* e4e4 associations with test positivity and mortality were little affected by excluding dementia and other *ApoE* e4-associated diagnoses reported before March 2017: future work should include recent preexisting diagnoses. More data are needed on *ApoE* and COVID-19 associations in other ancestry groups, as numbers of UKB participants of such groups are unfortunately too small for this analysis.

In conclusion, *ApoE* e4e4 genotype is associated with COVID-19 test positivity at genome-wide significance (ie, *p* < 5 × 10^−8^) in UKB, using data covering a longer period than previously reported. Similarly, the e4e4 genotype was associated with a 4-fold increase in mortality after testing positive for COVID-19, in UKB. Independent replications are needed to confirm our findings and mechanistic work is needed to understand how *ApoE* e4e4 results in the marked increase in vulnerability, especially for COVID-19 mortality. These findings also demonstrate that risks for COVID-19 mortality are not simply related to advanced chronological age or the comorbidities commonly seen in aging.

## Funding

C.L.K., L.C.P., G.A.K., and D.M. are supported in part by an R21 grant (R21AG060018) funded by National Institute on Aging, National Institutes of Health, USA. UK Medical Research Council award MR/S009892/1 (PI Melzer) supports J.L.A. J.A.H.M. is supported by National Institute for Health Research, UK Doctoral Research Fellowship DRF-2014-07-177. The views expressed in this publication are those of the author(s) and not necessarily those of the NHS, the National Institute for Health Research or the Department of Health.
